# Nuclear SOX9 Expression and Short-Term Survival Outcomes in Early-Stage Luminal Breast Cancer: An Immunohistochemical and Retrospective Cohort Analysis

**DOI:** 10.3390/ijms27188337

**Published:** 2026-09-19

**Authors:** Ebru Karci, Sabin Goktas Aydin, Osman Erinc, Taskin Erkin Uresin, Sevinc Dagistanli, Nilay Bakoglu Malinowski, Ahmet Aydın, Onur Tanrikulu

**Affiliations:** 1 Department of Medical Oncology, Faculty of Medicine, Istanbul Atlas University, 34408 Istanbul, Turkey; 2 Department of Medical Oncology, South Tees Hospitals NHS Foundation Trust, Middlesbrough TS4 3BW, UK; drsabingoktas@gmail.com; 3 Department of Internal Medicine, Kanuni Sultan Suleyman Training and Research Hospital, 34303 Istanbul, Turkey; 4 Department of Pathology, Kanuni Sultan Suleyman Training and Research Hospital, 34303 Istanbul, Turkey; taskinerkin.uresin@saglik.gov.tr; 5 Department of General Surgery, Kanuni Sultan Suleyman Training and Research Hospital, 34303 Istanbul, Turkey; sdagistanli2@hotmail.com; 6 Department of Pathology, Faculty of Medicine, İstanbul Medipol University, 34214 Istanbul, Turkey; nilay.bakoglu@medipol.edu.tr; 7 Department of Internal Medicine, Faculty of Medicine, İstanbul Medipol University, 34214 Istanbul, Turkey; uzm.dr.ahmetaydin@gmail.com

**Keywords:** SOX9, luminal breast cancer, immunohistochemistry, H-score, disease-free survival, overall survival, prognostic biomarker, cellular plasticity

## Abstract

SOX9 is a transcription factor linked to cellular plasticity and aggressive behavior in breast cancer. We investigated whether nuclear SOX9 expression correlates with short-term (5-year) survival outcomes in luminal breast cancer. In a retrospective cohort of 60 patients with estrogen receptor-positive invasive breast carcinoma, nuclear SOX9 expression was evaluated via immunohistochemical H-scores (0–300). Survival was analyzed continuously, by median-split dichotomy, and using a 1% nuclear-staining threshold via Kaplan–Meier and Cox regression models. Most tumors were luminal B (73.3%), and 45% were node-positive. Nuclear SOX9 was detected in 51.7% of cases (median positive H-score: 30). Over follow-up, 13 disease-free survival (DFS) and 12 overall survival (OS) events occurred. Continuous H-score did not correlate with clinicopathological variables, DFS (*p* = 0.576), or OS (*p* = 0.613). A median-split showed higher 5-year DFS for high-expression tumors (89.7% vs. 74.2%, *p* = 0.048), but this borderline protective trend was unconfirmed by Cox regression (*p* = 0.063) or the 1% threshold; ROC analysis showed no discrimination (AUC: 0.34–0.36). Standalone nuclear SOX9 expression has no significant impact on short-term (5-year) prognosis in early-stage luminal breast cancer; extended follow-up is warranted to assess potential late recurrence.

## 1. Introduction

Luminal-type breast cancer, characterized primarily by estrogen receptor (ER) positivity, represents the most common subtype of breast malignancy worldwide. In its early stages, luminal-type breast cancer patients generally undergo primary surgical resection, followed by adjuvant endocrine therapy, which is the most commonly used clinical method for long-term positive prognosis. Despite these therapeutic successes, a significant subset of these patients experience disease recurrence. This recurrence develops within the initial five years post-treatment or as late-stage distant metastases decades later [1,2]. This clinical paradox strongly suggests that the underlying tumor biology driving recurrence extends beyond classical proliferation indexes, requiring the identification of deeper molecular mechanisms that cause therapy evasion and late relapse [3].

Accumulating evidence suggests that conventional biomarkers of cell proliferation are insufficient to fully predict long-term clinical outcomes in early-stage luminal breast cancer. Instead, recent translational research shows the critical roles of cellular plasticity and the acquisition of a cancer stem cell (CSC)-like progenitor phenotype in mediating therapeutic failure [4,5]. These plastic cellular states enable a subpopulation of tumor cells to transition into a dedifferentiated, resilient state, allowing them to survive as minimal residual disease (MRD) under the selective pressure of adjuvant endocrine therapies [5,6]. Therefore, understanding the molecular master regulators that give rise to this phenotypic flexibility is critical to identifying patients at high risk for recurrence.

Among the key transcription factors governing cellular fate, SRY-box transcription factor 9 (SOX9) has emerged as a central player in both normal mammary gland development and breast oncogenesis [7]. While physiologically essential for embryonic development and lineage differentiation, the pathological upregulation of SOX9 has been shown to heavily affect cellular plasticity and progenitor cell maintenance in human breast epithelium [4,8]. Mechanistically, SOX9 interacts dynamically with key oncogenic networks, including the Wnt/β-catenin signaling pathway, to alter cell differentiation states, sustain stemness traits, and promote aggressive biological behavior [9]. Furthermore, high expression of SOX9 is increasingly linked to the development of endocrine resistance, serving as a functional driver that permits luminal cells to adapt and survive despite hormone deprivation therapies [10]. Notably, SOX9 has been shown to actively govern the luminal progenitor state within the normal mammary epithelial hierarchy [4], and functions as an epigenetic driver of anti-estrogen resistance through HDAC5-mediated nuclear stabilization that promotes tamoxifen tolerance in estrogen receptor-positive models [10]. These lineage-specific mechanisms provide direct biological rationale for evaluating nuclear SOX9 within luminal-type tumors specifically, rather than in the basal-like or triple-negative contexts in which SOX9 is more commonly studied.

Current clinical data correlate elevated SOX9 expression, whether nuclear or cytoplasmic, with poor prognostic features and decreased overall survival across various breast cancer subtypes [8,11]. However, these existing studies are heavily limited by their reliance on highly heterogeneous patient groups. There is a critical knowledge gap in understanding the specific, independent prognostic impact of SOX9 expression within homogeneous patient populations that particularly present with early-stage, luminal-type tumors managed with standard adjuvant endocrine therapy. Investigating SOX9 within a controlled clinical setting is crucial to analyze its direct impact as a predictive and prognostic biomarker.

To address this gap, this study evaluates a standardized set of early-stage luminal breast cancer patients who underwent primary surgical intervention and received adjuvant endocrine therapy (with or without companion chemotherapy) between 2019 and 2021. Utilizing immunohistochemistry (IHC) on archived formalin-fixed paraffin-embedded (FFPE) tumor tissues, we aimed to systematically quantify SOX9 expression levels and correlate these findings with 5-year disease-free survival (DFS) metrics. Through this approach, this study systematically quantifies nuclear SOX9 expression in a uniform cohort of early-stage, ER-positive luminal breast cancers to determine whether baseline nuclear SOX9 serves as a standalone prognostic biomarker for 5-year disease-free and overall survival.

## 2. Results

### 2.1. Patient and Tumor Characteristics

The study cohort comprised 60 women with estrogen receptor-positive (luminal) invasive breast carcinoma (Table 1). The mean age was 59.7 ± 13.4 years (median 56.5), and 46 patients (76.7%) were aged ≥50 years. Invasive ductal carcinoma was the predominant histological type (45 patients, 75.0%), followed by invasive lobular carcinoma (8, 13.3%). Most tumors were stage T2 (36, 62.1%), and 27 patients (45.0%) had pathologically node-positive disease. Progesterone receptor expression was positive in 49 patients (81.7%), and HER2 was negative in 47 (78.3%); Ki-67 was ≥15% in 40 tumors (67.8%). Accordingly, 44 tumors (73.3%) were classified as luminal B and 16 (26.7%) as luminal A. Adjuvant chemotherapy was administered to 31 patients (52.5%) and adjuvant endocrine therapy to the remainder, most commonly an aromatase inhibitor (40, 67.8%). During follow-up, 2 patients (3.3%) developed a recurrence and 12 (20.0%) died, yielding 13 disease-free survival (DFS) events and 12 overall survival (OS) events.

### 2.2. SOX9 Expression

**Figure 1:**
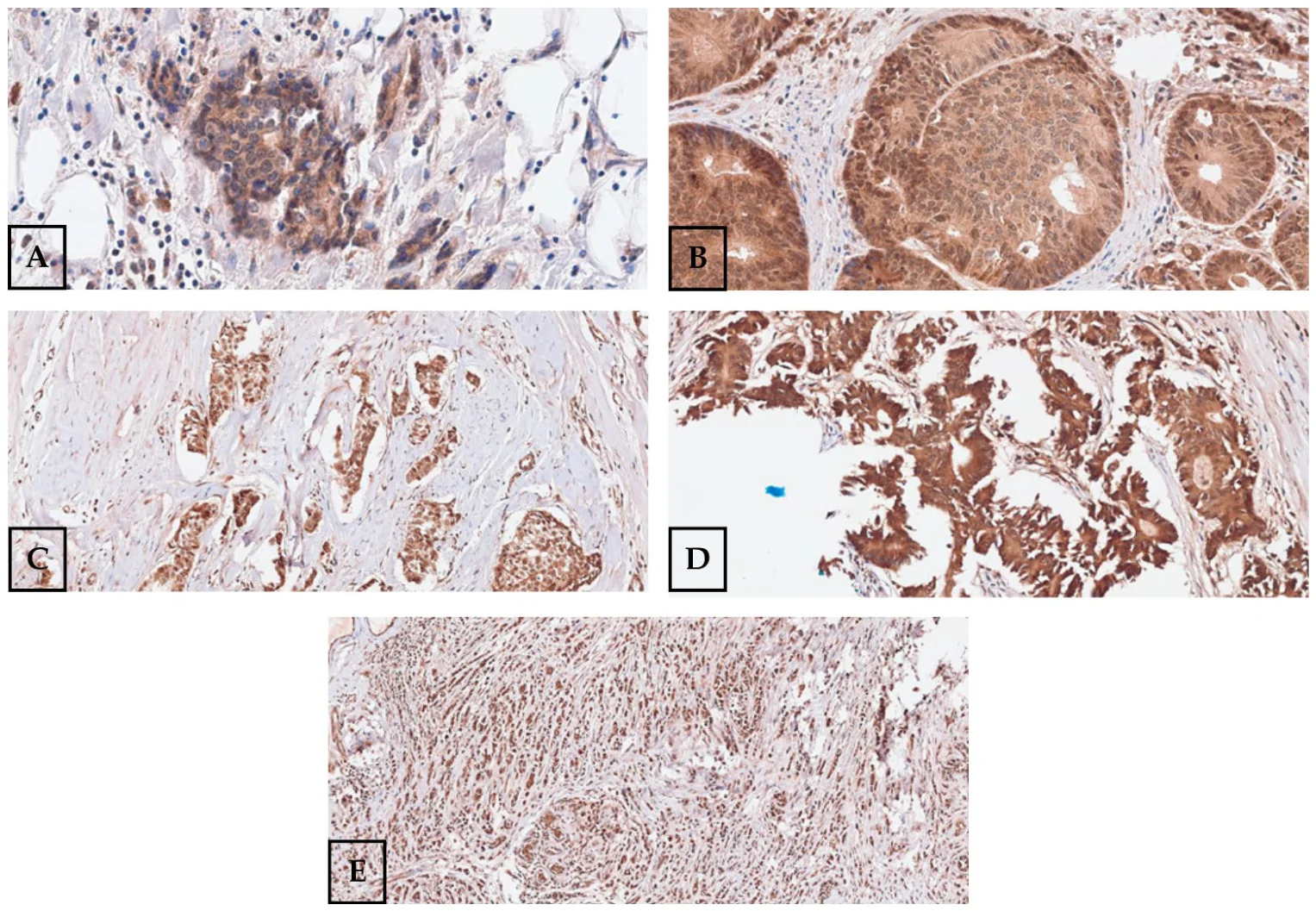
Representative immunohistochemical staining patterns of nuclear SOX9 across invasive luminal breast carcinoma lesions. (**A**) Negative SOX9 expression within a tumor nest, demonstrating no nuclear immunoreactivity. (**B**) Negative SOX9 expression in an in situ carcinoma component, showing a complete absence of nuclear staining with accompanying cytoplasmic reactivity, alongside nuclear positivity in background lymphocytes. (**C**) Weak and moderate nuclear SOX9 expression with accompanying cytoplasmic reactivity in a carcinoma tumor cell group. (**D**) Moderate, dual cytoplasmic and nuclear SOX9 positivity in a carcinoma exhibiting papillary architecture. (**E**) Strong and diffuse nuclear SOX9 positivity in an invasive lobular carcinoma (Immunohistochemistry Stain; Brown chromogenic signal indicates positive SOX9 staining; nuclei are counterstained blue with hematoxylin. panels (**A**,**C**) original magnification, ×400; panels (**B**–**E**) original magnification, ×200).

Nuclear SOX9 staining of any intensity was present in 31 tumors (51.7%); when a ≥1% nuclear-staining threshold was applied, 22 tumors (36.7%) were classified as SOX9-positive (Table 2). The median proportion of stained nuclei was 0.5% (range 0–100%). Among positive cases, the median H-score was 30 (interquartile range 1.5–154; overall range 0–300). Dichotomization at the cohort median H-score defined 29 high-expression and 31 low-expression tumors. Non-neoplastic stromal cells uniformly lacked nuclear SOX9 expression across all sections, confirming assay specificity. Representative immunohistochemical staining profiles illustrating the spectrum of nuclear SOX9 intensity (negative, weak, moderate, and strong) alongside focal cytoplasmic reactivity are shown in Figure 1.

### 2.3. SOX9 and Clinicopathological Features

The SOX9 H-score (high vs. low) was not significantly associated with any clinicopathological variable, including age, histological type, T stage, nodal status, progesterone receptor, HER2, Ki-67 or luminal subtype (all *p* > 0.05; Table 3). The largest, though non-significant, differences were observed for nodal status (node-positive disease in 34.5% of high-expression vs. 54.8% of low-expression tumors; *p* = 0.129) and mortality (10.3% vs. 29.0%; *p* = 0.107). No recurrence occurred in the high-expression group (0% vs. 6.5%; *p* = 0.492).

### 2.4. SOX9 and Disease-Free Survival

**Figure 2:**
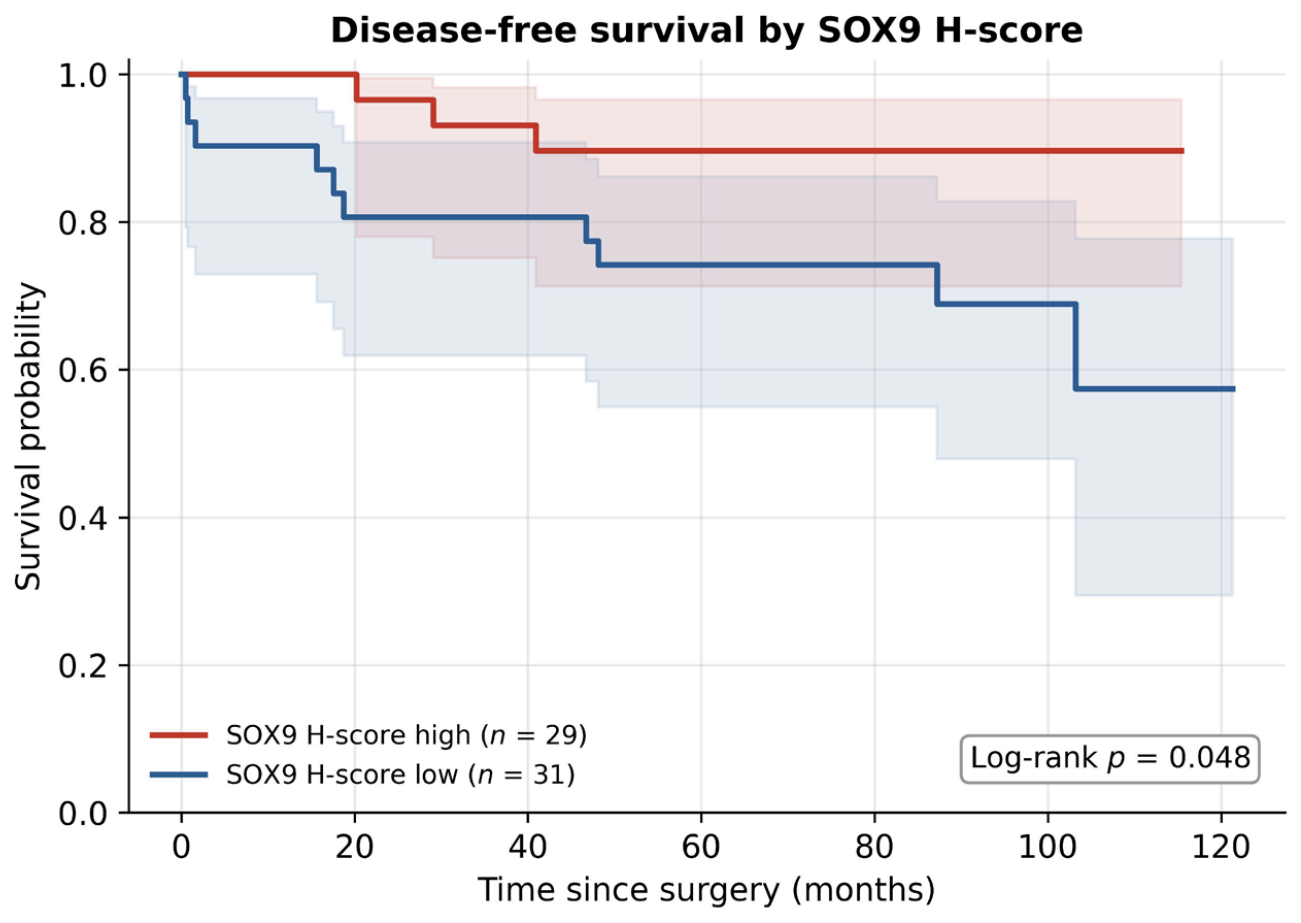
Disease-free survival stratified by SOX9 H-score (high vs. low, median split). Solid lines represent Kaplan–Meier survival estimates (red, SOX9 H-score high; blue, SOX9 H-score low), and the corresponding shaded areas denote the 95% confidence intervals. Groups were compared using the log-rank test.

On continuous analysis, the SOX9 H-score was not associated with DFS (hazard ratio [HR] per 10-unit increase, 0.98; 95% confidence interval [CI], 0.90–1.06; *p* = 0.576; Table 4). Using the median-based dichotomy, the 5-year DFS estimate was higher in the high-expression group than in the low-expression group (89.7% vs. 74.2%; log-rank *p* = 0.048; Figure 2). This borderline difference was in the direction of a protective rather than an adverse effect and was not corroborated by the corresponding Cox model (HR 0.29, 95% CI 0.08–1.07; *p* = 0.063) or by the ≥1% threshold (HR 0.53, 95% CI 0.14–1.92; *p* = 0.332; log-rank *p* = 0.324). All DFS events occurred within the Ki-67 ≥ 15% and luminal B strata, producing significant log-rank tests (*p* = 0.012 and *p* = 0.026, respectively; Table 5) but non-estimable Cox hazard ratios owing to complete separation. Node-positive disease (HR 2.05, 95% CI 0.67–6.29; *p* = 0.208) and higher T stage (HR 2.91, 95% CI 0.63–13.32; *p* = 0.169) showed non-significant trends toward worse DFS. In an exploratory multivariable model containing the SOX9 H-score and nodal status, neither variable was independently prognostic (H-score HR 0.98, *p* = 0.623; node-positive HR 2.02, *p* = 0.221).

### 2.5. SOX9 and Overall Survival

**Figure 3:**
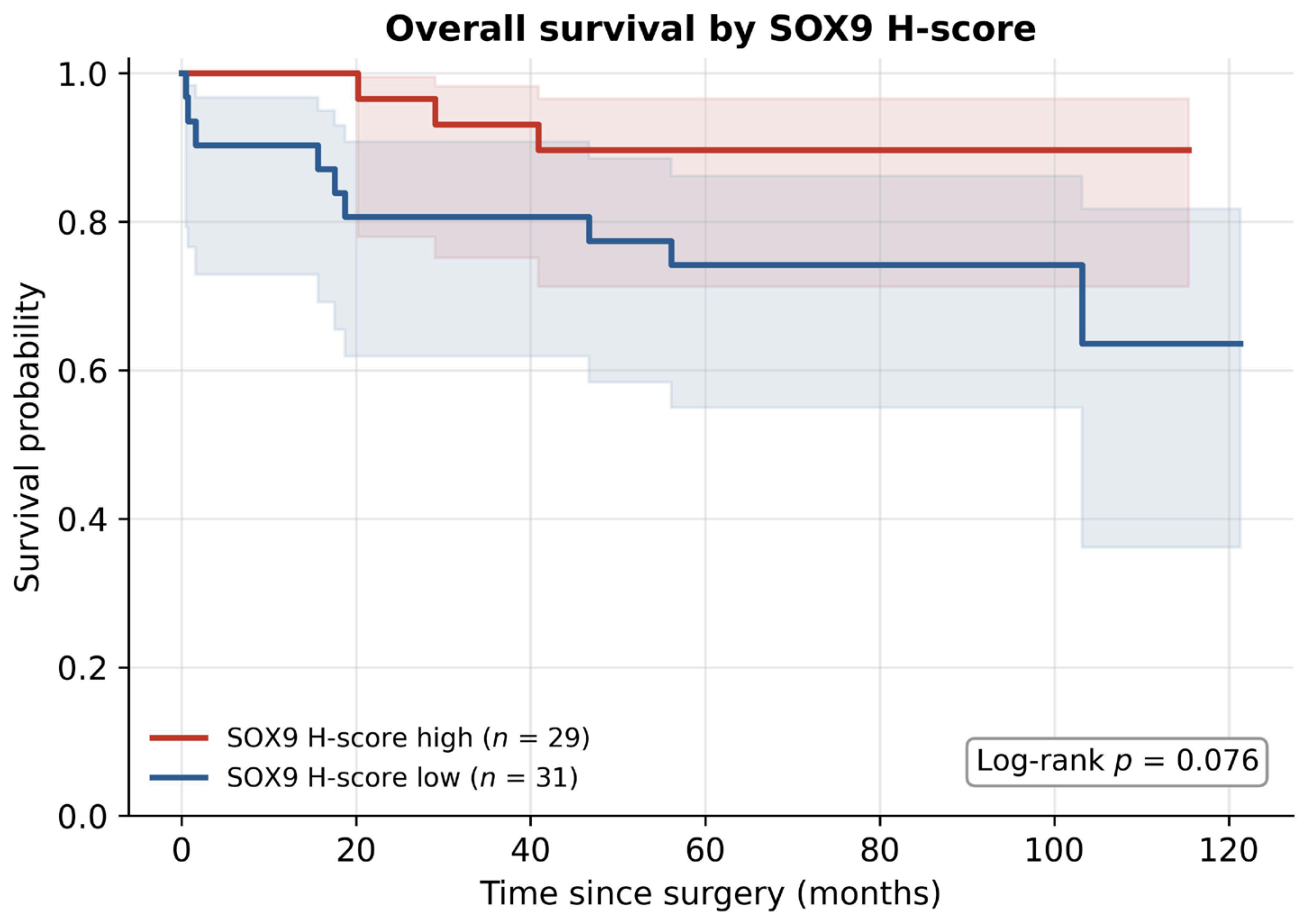
Overall survival stratified by SOX9 H-score (high vs. low, median split). Solid lines represent Kaplan–Meier survival estimates (red, SOX9 H-score high; blue, SOX9 H-score low), and the corresponding shaded areas denote the 95% confidence intervals. Groups were compared using the log-rank test.

Findings for OS paralleled those for DFS (Table 6 and Table 7; Figure 3). The continuous H-score was not associated with OS (HR per 10 units 0.98, 95% CI 0.90–1.06; *p* = 0.613). The 5-year OS estimate was again higher in the high-expression group than in the low-expression group (89.7% vs. 74.2%), but the difference did not reach significance (log-rank *p* = 0.076; Cox HR 0.32, 95% CI 0.09–1.20; *p* = 0.092). Ki-67 (*p* = 0.012) and luminal subtype (*p* = 0.031) separated the survival curves, whereas nodal status, T stage and age did not (all *p* > 0.05).

### 2.6. Discriminative Ability and Correlations

**Figure 4:**
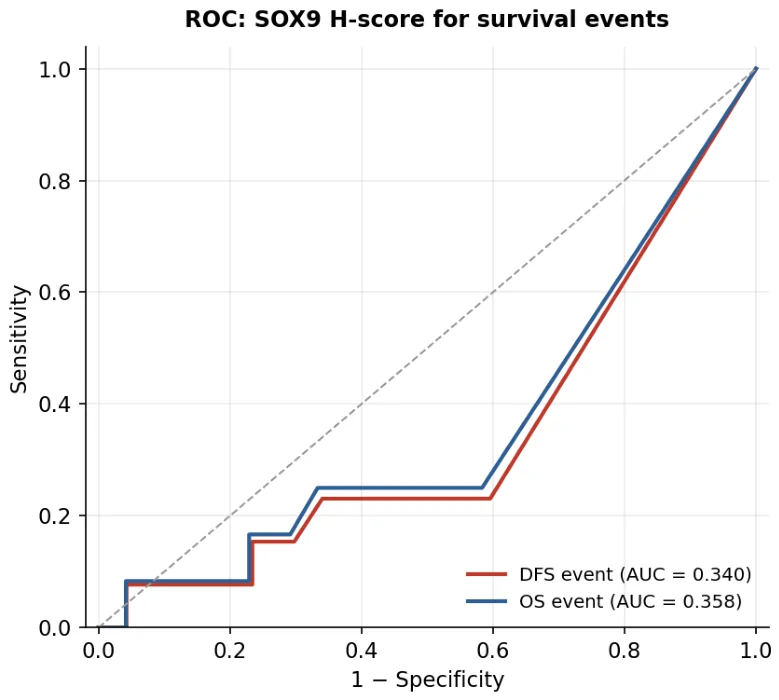
Receiver operating characteristic (ROC) curves of the SOX9 H-score for predicting survival events. Solid lines represent the ROC curves for disease-free survival (DFS) events (red) and overall survival (OS) events (blue); the dashed diagonal line indicates the reference line of no discrimination (AUC = 0.50). AUC, area under the curve.

**Figure 5:**
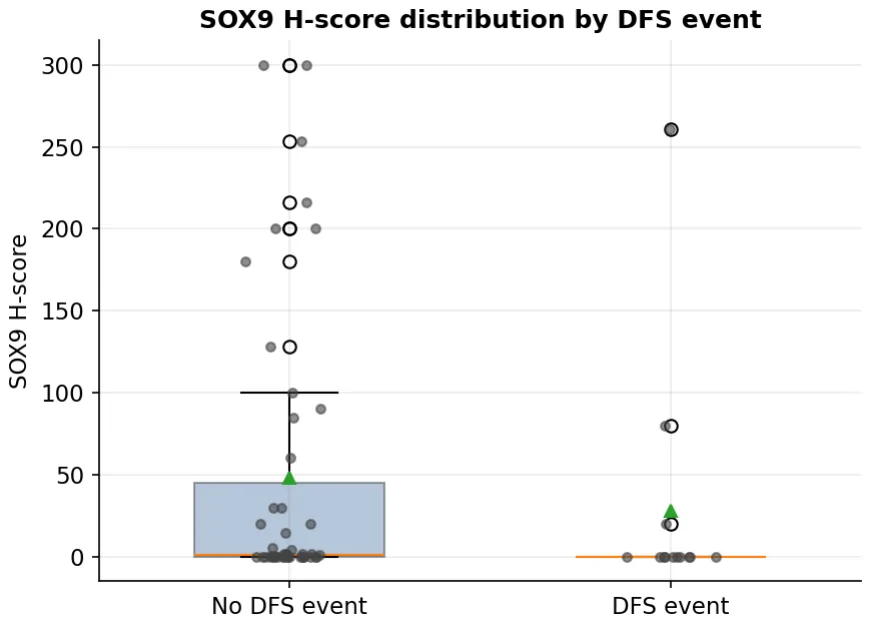
Distribution of SOX9 H-score in patients with vs. without a disease-free survival (DFS) event. Boxes represent the interquartile range (IQR), the orange horizontal line indicates the median, and the green triangle indicates the mean. Whiskers extend to 1.5 × IQR; open circles denote outliers beyond this range, and grey dots represent individual patient values.

ROC analysis confirmed that the SOX9 H-score did not discriminate between patients who experienced an event and those who did not, with areas under the curve of 0.34 for DFS and 0.358 for OS (both < 0.50; Table 8; Figure 4). Consistent with this, the H-score showed only weak, non-significant negative correlations with DFS time (Spearman r = −0.14; *p* = 0.291) and OS time (r = −0.16; *p* = 0.227), and no correlation with age or T stage (Table 9; Figure 5).

## 3. Discussion

The primary objective of this study was to evaluate the clinical and prognostic significance of nuclear SOX9 expression as a biomarker of cellular plasticity and treatment evasion in a highly uniform cohort of patients with early-stage, estrogen receptor-positive luminal invasive breast carcinoma treated with standard adjuvant endocrine therapy. Contrary to our initial hypothesis and the existing literature, which characterizes SOX9 as a universal master regulator of aggressive tumor progression and therapy resistance [8,12], our findings show that nuclear SOX9 expression is not an independent prognostic factor for either disease-free survival (DFS) or overall survival (OS) in this patient population. This observation parallels findings in other epithelial malignancies, where nuclear SOX9 expression alone showed no survival correlation, whereas cytoplasmic SOX9 mislocalization carried independent prognostic significance [13]—a pattern occasionally noted in our cohort alongside nuclear staining (Figure 1D).

Notably, the only nominally significant relation uncovered in our survival analyses was seen in the median-based difference in the H-score, which paradoxically revealed a higher 5-year DFS rate in the high-expression group compared to the low-expression group (89.7% vs. 74.2%). However, this borderline difference trended toward a protective direction, diverging from our initial hypothesis. This isolated finding was not confirmed by any other statistical approach. In fact, when SOX9 was evaluated as a continuous score, grouped by a 1% threshold, or tested in multivariable regression models, it showed no meaningful relationship with patient survival.

The borderline significant log-rank result (*p* = 0.048) favoring the high-SOX9 expression group present in our median-split analysis introduces an unusual biological paradox. Specifically, elevated nuclear SOX9 correlated with favorable 5-year DFS, rather than acting as an aggressive driver of recurrence and therapy resistance. Mechanistically, while SOX9 is mostly characterized as an oncogenic driver that maintains cancer stem cell (CSC) traits, new evidence suggests it can perform highly context-dependent roles. Depending on the specific microenvironment and cell lineage, it can act as a tumor suppressor [14,15]. In mature, estrogen receptor-positive luminal cells, higher baseline nuclear SOX9 might reflect a more stable or differentiated cellular state. That state can still be responsive to endocrine therapy, whereas complete absence of the marker could indicate a loss of baseline lineage control.

However, an alternative and statistically more plausible explanation lies in data dichotomization. Splitting a continuous variable like the H-score exactly at the cohort median is known to artificially alter statistical power. This splitting creates borderline significant results that disappear under more robust testing structures [16,17]. This situation is clearly observed in our data. When SOX9 was evaluated as a continuous variable or run through a multivariable Cox model alongside established features like lymph node status, any hint of a protective effect completely disappeared. Furthermore, our box plot analysis shows that the majority of patients who suffered a DFS event had an H-score of exactly zero, yet a few individuals without events also shared this baseline. Therefore, this nominal significance should be interpreted with extreme caution, which may reflect a statistical fluctuation rather than a protective mechanism of SOX9.

Previous clinical studies that show a strong correlation between elevated SOX9 expression and poor survival profiles have generally analyzed highly heterogeneous patient populations [18]. In such mixed cohorts, aggressive, poorly differentiated, and receptor-negative phenotypes, such as triple-negative breast cancer (TNBC) and HER2-enriched subtypes, often skew the survival metrics [19]. Mechanistically, in these aggressive environments, SOX9 operates as an active oncogenic engine that drives the epithelial–mesenchymal transition (EMT), suppresses extrinsic apoptotic pathways, and accelerates metastatic propagation [20,21]. Consequently, when high-grade, receptor-negative tumors with naturally high SOX9 levels are grouped together with lower-grade luminal tumors, the subtype-specific behavior of the marker is easily obscured. This leads to generalized conclusions that frame SOX9 as a universal marker of treatment failure, masking its true clinical implication [19].

In contrast, our study uniquely isolated a uniform, early-stage, estrogen receptor-positive luminal cohort under standard adjuvant endocrine control. Within this tightly bounded clinical framework, nuclear SOX9 expression was found to be exceptionally low and scattered, yielding a cohort median H-score of just 0.5. This lineage and microenvironmental divergence is highly critical. It shows the transcription factor networks regulating cellular plasticity operate under fundamentally distinct feedback loops in mature, hormone-dependent luminal cells compared to basal-like cells. While established preclinical data show that chronic upregulation of an SOX9-driven progenitor state can eventually lead to long-term tamoxifen and endocrine resistance [22], our clinical findings suggest that during the early post-treatment phase, low baseline nuclear SOX9 does not actively dictate early recurrence. This highlights that the role of SOX9 in cellular plasticity is highly subtype-dependent and cannot be reliably predicted from cohorts heterogeneous to early-stage luminal disease.

Several critical limitations must be acknowledged when interpreting the findings of this study. First, the retrospective, single-center design naturally introduces potential selection biases and limits the application of our observations to a wider population. Second, our analysis was constrained by a relatively small sample size of 60 patients. Furthermore, the occurrence of only 2 recurrences and 12 deaths during the active follow-up window resulted in a remarkably low clinical event rate. This shortage of statistics severely underpowered our survival models, making them highly sensitive to distribution fluctuations and less capable of identifying subtle hazards. Additionally, although slides were evaluated by two blinded pathologists using a consensus-based approach, independent individual scoring sheets were not retained prior to final consensus, preventing formal assessment of inter-observer reliability metrics.

In addition, the low event frequency caused a statistical issue known as complete separation in the Cox models for Ki-67 and luminal subtype. All DFS and OS events occurred exclusively within the Ki-67 ≥ 15% and Luminal B categories, whereas no events occurred in the reference groups. This stark difference made the hazard ratios mathematically unstable, and the ratios were stated as “Not Estimable” (NE) values [23].

Lastly, our 5-year timeline is a standard clinical benchmark. However, it represents a relatively short follow-up window because of the specific biology of estrogen receptor-positive breast cancers. Unlike aggressive triple-negative tumors, which tend to relapse rapidly, luminal tumors are characterized by a steady, persistent risk of late recurrence that can manifest 10, 20, or even 30 years after primary surgical resection [24]. Therefore, a much longer tracking period is required to rule out whether low-level nuclear SOX9 expression acts as a true latent driver of late-stage systemic relapse.

The lack of an independent prognostic connection between nuclear SOX9 and survival outcomes in our cohort highlights an emerging consensus in recent oncology literature. Our findings directly parallel contemporary solid tumor investigations, such as a 2024 study on primary colorectal cancer by Gutiérrez-Gil et al., which similarly utilized an H-score framework and demonstrated that SOX9 expression was entirely independent of aggressive clinicopathological features like lymph node metastasis [25]. Consistent with these observations, the absence of an independent prognostic association in our cohort indicates that standalone nuclear SOX9 assessment does not provide incremental risk stratification beyond established clinicopathological parameters.

In clinical practice, the Ki-67 proliferation index and consensus luminal subtyping remain the definitive parameters for evaluating recurrence risk and guiding therapeutic escalation [26,27]. Future translational investigations should evaluate composite biomarker panels integrating SOX9 with downstream regulatory axes, including Wnt/β-catenin and Slug, across multi-institutional cohorts with extended follow-up windows.

In conclusion, although preclinical models link SOX9 to endocrine resistance, our clinical data indicate that standalone nuclear SOX9 expression does not predict 5-year survival or recurrence in early-stage luminal breast cancer. The nominal protective trend observed in our median-split analysis is fragile and likely represents a statistical artifact rather than a true biological effect. Consequently, standard parameters, such as the Ki-67 index and luminal subtyping, remain the definitive choices for immediate risk classification. Ultimately, these findings warn against generalizing biomarker functions across completely different breast cancer subtypes, highlighting the need for multi-marker evaluations in future research.

## 4. Materials and Methods

This retrospective biomarker study was designed and reported in accordance with the Reporting Recommendations for Tumor Marker Prognostic Studies (REMARK) guidelines [28]. In this study, archived tumor tissue samples were re-examined via immunohistochemical staining. A total of 100 female patients, aged 18 to 75, who were diagnosed with early-stage breast cancer between January 2019 and December 2021, were screened for the study. To be included in the study, patients needed a histopathologically confirmed diagnosis of invasive breast carcinoma, documented estrogen receptor (ER) positivity via immunohistochemistry indicating a luminal subtype, and a clinical history of primary surgery followed by adjuvant endocrine therapy. The administration status of adjuvant chemotherapy was recorded for subsequent subgroup analyses. On the other hand, patients were excluded from the study if they had received neoadjuvant systemic therapy, presented with distant metastatic disease at the time of initial diagnosis, displayed insufficient archival paraffin block material, or lacked regular and documented clinical follow-up data.

Patient demographics and tumor characteristics were retrieved from the institutional electronic medical record system and pathology archives. The collected dataset included patient age, menopausal status, total tumor size, histological subtype, and histological grade. Also, lymph node status, the presence or absence of lymphovascular invasion, the Ki-67 proliferation index, and the specific adjuvant therapeutic methods administered to each patient were recorded. Surrogate intrinsic subtypes were defined according to St. Gallen consensus recommendations [26] and International Ki-67 Working Group criteria [29]: Luminal A (ER-positive, HER2-negative, PR ≥ 20%, and Ki-67 < 15%) and Luminal B (ER-positive with Ki-67 ≥ 15%, HER2-positive/equivocal, or PR < 20%) [26,29].

Immunohistochemical (IHC) analysis was performed on 4-micrometer-thick sections obtained from archived formalin-fixed paraffin-embedded (FFPE) tumor tissue blocks. Sections were deparaffinized in xylene and rehydrated through a graded ethanol series. Heat-induced antigen retrieval was conducted in EDTA buffer (pH 9.0) using a pressure cooker for 20 min. Endogenous peroxidase activity was inactivated by incubation in 3% hydrogen peroxide for 10 min. Primary antibody incubation was carried out using a rabbit monoclonal anti-SOX9 antibody (clone EP317; Epitomics, Burlingame, CA, USA; catalog no. AC-0284RUO) at a 1:500 dilution for 60 min at room temperature. Signal detection was performed using the UltraVision LP HRP Polymer Detection System (Thermo Fisher Scientific, Fremont, CA, USA; catalog no. TL-015-HD) with 3,3′-diaminobenzidine (DAB) as the chromogen. Sections were counterstained with Gill’s hematoxylin, dehydrated, and cover-slipped. Adjacent normal terminal duct lobular units were utilized as internal positive controls, while non-neoplastic stromal cells (fibroblasts, endothelial cells, and infiltrating lymphocytes) within the tumor microenvironment served as internal negative controls.

The evaluation of nuclear SOX9 was conducted semi-quantitatively by two independent pathologists blinded to patient clinical outcomes and demographics. Staining was evaluated using a consensus review protocol to establish a unified H-score for each case; because a single agreed-upon consensus score was recorded directly in the study database, independent pre-consensus ratings were not archived, precluding formal inter-observer concordance calculations (Cohen’s kappa or ICC). All financial costs associated with the staining procedures and pathological evaluations were funded directly by the principal investigators.

### Statistical Analysis

The analysis cohort comprised 60 patients with ER-positive (luminal) invasive breast carcinoma. SOX9 nuclear expression was quantified as an H-score (0–300) [30], calculated as the percentage of positive nuclei multiplied by the mean staining intensity (1–3) derived from the strong/moderate/weak intensity distribution. SOX9 was analyzed as a continuous H-score, as an H-score-based dichotomy (high vs. low, split at the cohort median of 0.5), and using a ≥1% nuclear-staining threshold. Disease-free survival (DFS) was defined as the time from surgery to recurrence or death from any cause and overall survival (OS) as the time from surgery to death from any cause; event-free patients were censored at last contact. Axillary nodal status reflected pathological node positivity. Categorical variables were compared with the Fisher exact or chi-square test and continuous variables with the Mann–Whitney U test. Survival was estimated by the Kaplan–Meier method with the log-rank test, and hazard ratios (HRs) were obtained from Cox proportional-hazard models. Given the event count (13 DFS and 12 OS events) and adhering to the events-per-variable (EPV) principle, multivariable Cox regression was restricted to a bivariate model incorporating the SOX9 H-score and pathological axillary nodal status. Ki-67 and luminal subtype were not included in multivariable Cox models due to zero events occurring in the reference strata (complete mathematical separation). The discriminative ability of the SOX9 H-score for survival events was assessed by ROC analysis. A post hoc power calculation indicated that with *n*= 60 patients and 13 DFS events (12 OS events), assuming a two-sided α = 0.05 and 80% statistical power, the study was powered to reliably detect a minimum hazard ratio (MDHR) of approximately ≥4.5 (or ≤0.22). Two-sided *p* < 0.05 was considered statistically significant. Analyses were performed in Python Software Foundation) using the pandas (v2.3.3), lifelines (v0.30.3), scikit-learn (v1.8.0), and scipy (v1.17.1) packages).

## Figures and Tables

**Table 1:** Clinicopathological characteristics of the study cohort (*n* = 60).

Characteristic	Category	*n* (%) or Mean ± SD
Age, years	Mean ± SD	59.7 ± 13.4
	Median (Q1–Q3)	56.5 (50–66.5)
Age group	<50	14 (23.3)
	≥50	46 (76.7)
Histological type	IDC	45 (75)
	ILC	8 (13.3)
	Other	7 (11.7)
T stage	T1	18 (31)
	T2	36 (62.1)
	T3	4 (6.9)
Axillary nodal status	Negative	33 (55)
	Positive	27 (45)
PR	Positive	49 (81.7)
	Negative	11 (18.3)
HER2	Negative	47 (78.3)
	Positive/equivocal	13 (21.7)
Ki-67	<15%	19 (32.2)
	≥15%	40 (67.8)
Luminal subtype	Luminal A	16 (26.7)
	Luminal B	44 (73.3)
Neoadjuvant therapy	Yes	1 (1.7)
	No	59 (98.3)
Adjuvant chemotherapy	Yes	31 (52.5)
	No	28 (47.5)
Adjuvant endocrine	Tamoxifen	19 (32.2)
	AI	40 (67.8)
Recurrence	Yes	2 (3.3)
Mortality		12 (20)

SD, standard deviation; Q1–Q3, interquartile range; IDC, invasive ductal carcinoma; ILC, invasive lobular carcinoma; PR, progesterone receptor; AI, aromatase inhibitor. Percentages are based on non-missing observations; some categories sum to <60 owing to missing data.

**Table 2:** SOX9 immunohistochemical expression (*n* = 60).

SOX9 Parameter	Category/Metric	Value
Nuclear status (any staining)	Positive	31 (51.7)
	Negative	29 (48.3)
Nuclear status (≥1% threshold)	SOX9+	22 (36.7)
	SOX9−	38 (63.3)
Nuclear staining (%)	Median (min–max)	0.5 (0–100)
H-score, whole cohort	Median (min–max)	0.5 (0–300)
H-score, positive cases	Median (Q1–Q3)	30 (1.5–154)
H-score dichotomy (median split)	High/Low	29/31

H-score (range 0–300) = percentage of positive nuclei × mean intensity (1–3). The median-split cutoff was an H-score of 0.5, which corresponds in practice to any detectable nuclear staining.

**Table 3:** Association between SOX9 H-score (high vs. low) and clinicopathological variables.

Variable	Category	H-Score High	H-Score Low	*p*
Age	mean ± SD	57.2 ± 9.9	62.0 ± 15.7	0.225 ^MWU^
Histology	IDC	22 (75.9)	23 (74.2)	0.952
	ILC	4 (13.8)	4 (12.9)	
	Other	3 (10.3)	4 (12.9)	
T stage	T1	10 (35.7)	8 (26.7)	0.741
	T2	16 (57.1)	20 (66.7)	
	T3–T4	2 (7.1)	2 (6.7)	
Nodal status	Negative	19 (65.5)	14 (45.2)	0.129
	Positive	10 (34.5)	17 (54.8)	
PR	Negative	5 (17.2)	6 (19.4)	1
	Positive	24 (82.8)	25 (80.6)	
HER2	Negative	24 (82.8)	23 (74.2)	0.536
	Positive/equivocal	5 (17.2)	8 (25.8)	
Ki-67	<15%	10 (34.5)	9 (30.0)	0.785
	≥15%	19 (65.5)	21 (70.0)	
Luminal subtype	Luminal A	9 (31.0)	7 (22.6)	0.563
	Luminal B	20 (69.0)	24 (77.4)	
Adjuvant chemo	Yes	14 (48.3)	17 (56.7)	0.606
Recurrence	Yes	0 (0.0)	2 (6.5)	0.492
Mortality	Ex	3 (10.3)	9 (29.0)	0.107

Data are *n* (%) except the age. MWU: Mann–Whitney U. No clinicopathological variable was significantly associated with SOX9 H-score (lowest *p* = 0.129 for nodal status).

**Table 4:** Cox regression for disease-free survival (events = 13/60).

Variable	Univariable HR (95% CI)	*p*	Multivariable HR (95% CI)	*p*
SOX9 H-score (per 10 units)	0.98 (0.90–1.06)	0.576	0.98 (0.90–1.07)	0.623
SOX9 H-score high vs. low	0.29 (0.08–1.07)	0.063	—	—
SOX9 ≥ 1% vs. <1%	0.53 (0.14–1.92)	0.332	—	—
Age ≥ 50 vs. <50	0.97 (0.26–3.55)	0.962	—	—
T3–T4 vs. T1–T2	2.91 (0.63–13.32)	0.169	—	—
Node-positive vs. negative	2.05 (0.67–6.29)	0.208	2.02 (0.66–6.19)	0.221
Ki-67 ≥ 15% vs. <15%	NE *	0.995	—	—
Luminal B vs. A	NE *	0.995	—	—

HR, hazard ratio; CI, confidence interval; NE, not estimable. * Complete mathematical separation occurred for Ki-67 (<15%) and Luminal subtype (Luminal A) due to zero events in reference categories, rendering Cox estimates unstable (evaluated via log-rank tests in Table 5). The multivariable model was restricted to SOX9 H-score and nodal status in accordance with events-per-variable (EPV) constraints.

**Table 5:** Kaplan–Meier disease-free survival by subgroup.

Subgroup	*n*	3 yr%	5 yr%	Median (Month) Log-Rank *p*
H-score high	29	93.1	89.7	NR *p* = 0.048
H-score low	31	80.6	74.2	NR
SOX9 < 1%	38	84.2	78.9	NR *p* = 0.324
SOX9 ≥ 1%	22	90.9	86.4	NR
Ki-67 ≥ 15%	40	80	72.5	NR *p* = 0.012
Ki-67 < 15%	19	100	100	NR
Luminal B	44	81.8	75	NR *p* = 0.026
Luminal A	16	100	100	NR
Node-negative	33	87.9	87.9	NR *p* = 0.199
Node-positive	27	85.2	74.1	NR
T2	36	86.1	80.6	NR *p* = 0.284
T3–T4	4	75	50	46.7 month
T1	18	88.9	88.9	NR

NR: not reached. Significant log-rank tests: Ki-67 (*p* = 0.012), luminal subtype (*p* = 0.026) and the SOX9 H-score median split (*p* = 0.048, high group favored). All DFS events occurred in the Ki-67 ≥ 15% and Luminal B strata. The SOX9 median-split result is borderline and not corroborated by the continuous H-score or Cox analysis (Table 4); the ≥1% threshold was non-significant.

**Table 6:** Cox regression for overall survival (events = 12/60).

Variable	Univariable HR (95% CI)	*p*	Multivariable HR (95% CI)	*p*
SOX9 H-score (per 10 units)	0.98 (0.90–1.06)	0.613	0.98 (0.90–1.07)	0.655
SOX9 H-score high vs. low	0.32 (0.09–1.20)	0.092	—	—
SOX9 ≥ 1% vs. <1%	0.57 (0.15–2.10)	0.397	—	—
Age ≥ 50 vs. <50	1.51 (0.33–6.92)	0.593	—	—
T3–T4 vs. T1–T2	3.23 (0.69–15.09)	0.135	—	—
Node-positive vs. -negative	1.73 (0.55–5.46)	0.349	1.70 (0.54–5.37)	0.368
Ki-67 ≥ 15% vs. <15%	NE *	0.995	—	—
Luminal B vs. A	NE *	0.995	—	—

HR, hazard ratio; CI, confidence interval; NE, not estimable. * Complete mathematical separation occurred for Ki-67 (<15%) and luminal subtype (Luminal A) due to zero events in the reference categories, rendering Cox estimates unstable (evaluated via log-rank tests in Table 7). The multivariable model was restricted to the SOX9 H-score and nodal status in accordance with events-per-variable (EPV) constraints.

**Table 7:** Kaplan–Meier overall survival by subgroup.

Subgroup	*n*	3 yr%	5 yr%	Median (Month) Log-Rank *p*
H-score high	29	93.1	89.7	NR *p* = 0.076
H-score low	31	80.6	74.2	NR
SOX9 < 1%	38	84.2	78.9	NR *p* = 0.39
SOX9 ≥ 1%	22	90.9	86.4	NR
Ki-67 ≥ 15%	40	80	72.4	NR *p* = 0.012
Ki-67 < 15%	19	100	100	NR
Luminal B	44	81.8	74.9	NR *p* = 0.031
Luminal A	16	100	100	NR
Node-negative	33	87.9	87.9	NR *p* = 0.343
Node-positive	27	85.2	74.1	NR
T2	36	86.1	80.5	NR *p* = 0.266
T3-T4	4	75	50	46.7 month
T1	18	88.9	88.9	NR

NR: not reached. Significant log-rank tests: Ki-67 (*p* = 0.012) and luminal subtype (*p* = 0.031). The SOX9 H-score high group showed a non-significant trend toward better OS (*p* = 0.076).

**Table 8:** ROC analysis of SOX9 H-score for survival events.

Endpoint	AUC	Optimal Cutoff	Interpretation
DFS event	0.34	H-score 261	No discrimination (AUC < 0.50)
OS event	0.358	H-score 261	No discrimination (AUC < 0.50)

AUC, area under the curve. AUC values below 0.50 indicate that the SOX9 H-score does not discriminate between patients who experienced an event and those who did not.

**Table 9:** Spearman correlations of the SOX9 H-score.

Variable	Spearman r	*p*
Age	−0.2	0.128
Tumor stage (T)	−0.03	0.824
DFS time (months)	−0.14	0.291
OS time (months)	−0.16	0.227

No correlation reached statistical significance; weak negative trends were observed between the SOX9 H-score and DFS/OS time.

## Data Availability

The data presented in this study are available upon request from the corresponding author. The data are not publicly available due to ethical restrictions.

## Abbreviations

The following abbreviations are used in this manuscript:

AI	Aromatase Inhibitor
AUC	Area Under the Curve
CI	Confidence Interval
CSC	Cancer Stem Cell
DFS	Disease-Free Survival
EMT	Epithelial–Mesenchymal Transition
ER	Estrogen Receptor
FFPE	Formalin-Fixed Paraffin-Embedded
HER2	Human Epidermal Growth Factor Receptor 2
HR	Hazard Ratio
IDC	Invasive Ductal Carcinoma
IHC	Immunohistochemistry/Immunohistochemical
ILC	Invasive Lobular Carcinoma
M-W U	Mann–Whitney U
MRD	Minimal Residual Disease
NE	Not Estimable
NR	Not Reached
OS	Overall Survival
PR	Progesterone Receptor
Q1–Q3	Interquartile Range (First to Third Quartile)
ROC	Receiver Operating Characteristic
SD	Standard Deviation
SOX9	SRY-Box Transcription Factor 9
TNBC	Triple-Negative Breast Cancer
